# Structural Characterization and Anti-HSV-1 and HSV-2 Activity of Glycolipids from the Marine Algae *Osmundaria obtusiloba* Isolated from Southeastern Brazilian Coast

**DOI:** 10.3390/md10040918

**Published:** 2012-04-23

**Authors:** Lauro M. de Souza, Guilherme L. Sassaki, Maria Teresa Villela Romanos, Eliana Barreto-Bergter

**Affiliations:** 1 Department of Biochemistry and Molecular Biology, Federal University of Paraná, Curitiba 81531-990, PR, Brazil; Email: laurosouza@hotmail.com (L.M.S.); sassaki@ufpr.br (G.L.S.); 2 Department of Virology , Institute of Microbiology, Federal University of Rio de Janeiro, Rio de Janeiro 21941-902, RJ, Brazil; Email: teresa.romanos@micro.ufrj.br; 3 Department of General Microbiology, Federal University of Rio de Janeiro, Rio de Janeiro 21941-902, RJ, Brazil

**Keywords:** *Osmundaria obtusiloba*, red alga, glycolipids, sulfoquinovosyldiacylglycerol, anti-herpes simplex activity

## Abstract

Glycolipids were extracted from the red alga *Osmundaria obtusiloba *from Southeastern Brazilian coast. The acetone insoluble material was extracted with chloroform/methanol and the lipids, enriched in glycolipids, were fractionated on a silica gel column eluted with chloroform, acetone and then methanol. Three major orcinol-positive bands were found in the acetone and methanol fractions, being detected by thin layer chromatography. The structures of the corresponding glycolipids were elucidated by ESI-MS and ^1^H/^13^C NMR analysis, on the basis of their tandem-MS behavior and HSQC, TOCSY fingerprints. For the first time, the structure of sulfoquinovosyldiacylglycerol from the red alga *Osmundaria obtusiloba* was characterized. This molecule exhibited potent antiviral activity against HSV-1 and HSV-2 with EC_50_ values of 42 µg/mL to HSV-1 and 12 µg/mL to HSV-2, respectively. Two other glycolipids, mono- and digalactosyldiacylglycerol, were also found in the alga, being characterized by ESI-MS/MS. The structural elucidation of algae glycolipids is a first step for a better understanding of the relation between these structures and their biological activities.

## 1. Introduction

Glycolipids constitute an important class of membrane lipids that are synthesized by both prokaryotic and eukaryotic organisms. Major glycolipids such as monogalactosyldiacylglycerol (MGDG), digalactosyldiacylglycerol (DGDG) and sulfoquinovosyldiacylglycerol (SQDG), along with phospholipids were isolated from red, green and brown algae, such as, for example, *Anfeltia tobuchiensis *(Rhodophyta), *Ulva fenestrate *(Chlorophyta), *Laminaria japonica *(Chlorophyta), *Sargassum pallidum* (Phaeophyta) [1], *Sargassum thunbergii* (Phaeophyta) [2], *Exophlyllum wentii* (Rhodophyta) [3] and *Chondria armata* (Rhodophyta) [4], as well as in simbiotic organisms having mycroalgae [5,6]. These lipids are reported to exhibit diverse biological functions [7,8]. MGDGs from the green alga *Chlorella vulgaris*, exhibit anti-tumor effect [9]. SQDG from the red alga *Gigartina tenella* inhibits the eukaryotic DNA polymerase and HIV-reverse transcriptase type 1 [10] and SQDG identified in *Porphyridium purpureum* and other microalgae presents antivirus activity [11]. Most studies show the antiviral activity of sulfated polysaccharides from marine alga [12,13,14,15,16,17,18] but studies using sulfoglycolipids have received less attention [19,20,21,22]. Due to economic importance of algae, as a rich source of bioactive compounds with potential biomedicinal interest, there is an increasing need for improved techniques for isolation pure compounds. In our search for potentially useful bioactive molecules of marine origin, we have isolated and identified the glycolipids from the red alga *Osmundaria obtusiloba* from Southeastern Brazilian coast using electrospray ionization tandem mass spectrometry (ESI-MS/MS) complemented with NMR. In the present study we also evaluated the inhibitory activity of sulfoquinovosyldiacylglycerol from the red marine alga *Osmundaria obtusiloba* on the herpes simplex virus type 1 (HSV-1) and type 2 (HSV-2). Herpes simplex viruses are responsible for a broad range of human infectious diseases. Despite acyclovir is the drug of choice for treating the HSV infections, new antiviral agents exhibiting different mechanisms of action is urgently needed, mainly due to the high number of acyclovir-resistant strains [23].

## 2. Results and Discussion

### 2.1. Lipid Fractionation

**Figure 1:**
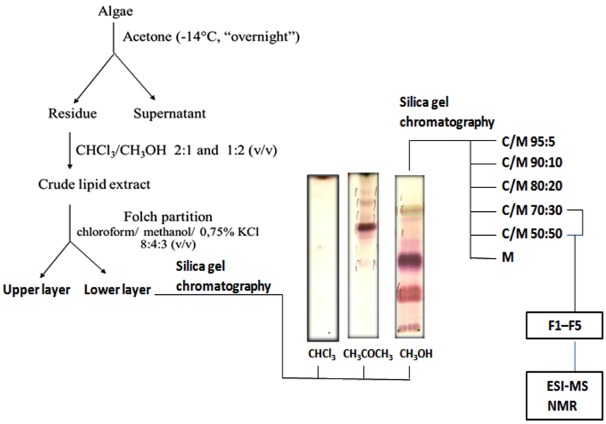
Overview of the strategy used for purification of glycolipids from *Osmundaria obtusiloba*. Purified or partially purified fractions are usually resolved by TLC and visualized with iodine and orcinol-H_2_SO_4_.

*Osmundaria obtusiloba* was treated with acetone and kept overnight at −14 °C. The acetone insoluble material was successively extracted with chloroform/methanol 2:1 and 1:2 (v/v) at room temperature [22,24]. The combined extracts were concentrated in vacuo and the crude lipid fraction was partitioned according to Folch and coworkers [25]. The lipids recovered from the Folch lower layer, enriched in glycolipids, were fractionated on a silica gel column, eluted with chloroform, acetone and then methanol. Figure 1 shows the steps of purification.

Orcinol reactive bands were detected on TLC from fractions eluted with acetone and methanol. A single main spot orcinol-positive was detected in the acetone fraction (F-Ac), whereas at least three positive spots could be visualized on TLC of methanol fraction (F-MeOH). This enriched glycolipid fraction was then purified by a second silica gel chromatography, eluted by a gradient of CHCl_3_/MeOH, increasing the solvent polarity, giving rise to fractions F1–F5 (Figure 1). These fractions were analyzed on TLC plates developed with CHCl_3_:MeOH:2M NH_4_OH (40:10:1 v/v/v) and visualized through iodine vapor and by spraying the plates with orcinol-sulfuric acid [26]. The partially purified glycolipids were then analyzed by ESI-MS in the positive and negative ionization modes.

### 2.2. Mass Spectrometry of Neutral Glycolipids

**Figure 2:**
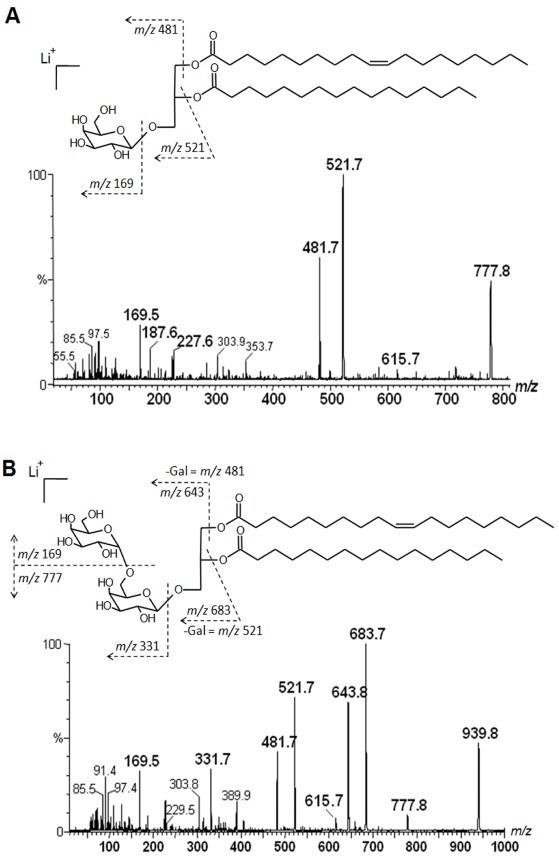
(**A**) Tandem-MS and fragmentation profile obtained from lithiated monogalactosyldiacylglycerol, precursor ion at *m/z* 777, from acetone Fraction (F-Ac); (**B**) from lithiated digalactosyldiacylglycerol, precursor ion at *m/z *939, from Fraction 5.

In order to improve the positive ion detection of neutral lipids, Li^+^ (LiCl) was added in the sample solvent, giving the molecules as lithiated ions. The acetone fraction (F-Ac) gave in MS^1^ an ion with *m/z* 777 [M + Li]^+^. This was fragmented by CID-MS, giving rise to characteristic spectrum of neutral glyceroglycolipids, with a fragment-ion at *m/z* 615, consistent with a monosaccharide residue loss, those at *m/z* 521 and 481 were consistent with loss of a palmitc acid (C_16:0_) and an unusual nonadecenoic (C_19:1_) acid, respectively. The fragment-ions from glycan moiety appeared at *m/z* 227, 187 and 169 (Figure 2A), thus being consistent with the fragmentation partner of a monogalactosyldiacylglycerol (MGDG) [27].

Fraction 5, an orcinol-positive sub-fraction from F-MeOH, was also analyzed in positive ion mode assisted by Li^+^, giving a main ion at *m/z* 939. These were fragmented giving rise to fragments at *m/z* 777 and a small *m/z* 615, consistent with loss a monosaccharide and a disaccharide, respectively. The fragments at *m/z* 683 and 643 were consistent with loss of a palmitic and a nonadecenoic acid, respectively. The fragments at *m/z* 389, 331, 187 and 169 confirmed the presence of a disaccharide, being this glycolipid consistent with a digalactosyldiacylglycerol (DGDG) [27,28]. Other fragments are shown in Figure 2B.

### 2.3. Mass Spectrometry of Sulfolipids

**Figure 3:**
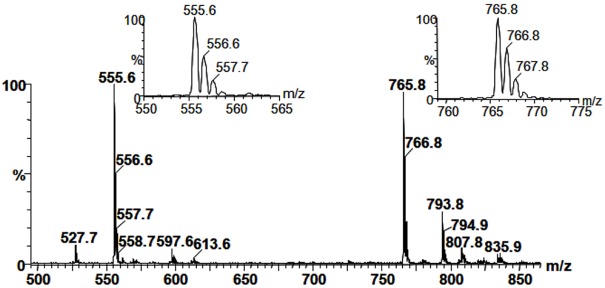
Spectrum from MS^1^ obtained in negative ionization mode from Fraction 1. The ion at *m/z* 555 is compatible with the structure of a sulfoquinovosylmonoacylglycerol, whereas the one at *m/z* 765, with sulfoquinovosyldiacylglycerol.

Fraction 1, a sub-fraction from F-MeOH, gave two positive spots for carbohydrate with lower *R_f_* values. Furthermore, the spectrum obtained in negative MS^1^ exhibited two main deprotonated ions with *m/z* 555 and 765 [M − H]^−^ (Figure 3).

The distribution of isotopologues was 10:4:1.5 (for *m/z* 555, 556 and 557, respectively) and 10:5:2 (for *m/z* 765, 766 and 767). The high intensity of the heavy isotopologues at *m/z* 557 and 767 is an indicative for the presence of a sulfur element in the molecules [29], occurring because of the presence of >4% of the heavy isotope (^34^S). In lipid fractions from vegetable sources, the presence of sulfur element added to positive result for carbohydrate strongly suggests the presence of sulfolipids, such as sulfoquinovosyldiacylglycerol (SQDG), a lipid associated with phototrophic organisms [30,31].

**Figure 4:**
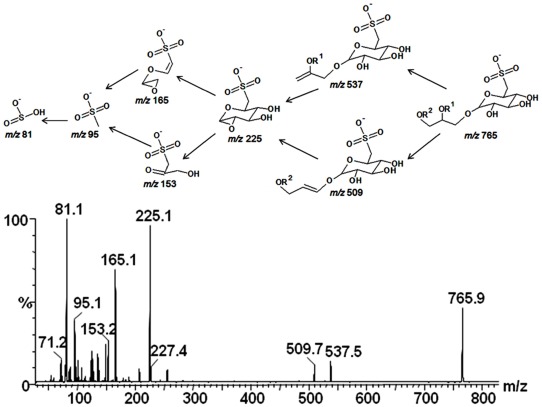
Tandem-MS fingerprint and a proposed fragmentation pathway of sulfoquinovosyldiacylglycerol, precursor ion at *m/z* 765, under collision induced dissociation-mass spectrometry.

In order to confirm the structures, the ions at *m/z* 555 and 765 were fragmented by the second stage tandem-MS. The results were consistent with the structure of the sulfoquinovosylmonoacylglycerol (SQMG-*m/z* 555) with a palmitic acid (C_16:0_), as indicated by the presence of fragments at *m/z* 299, 243, 225, 165, 153, 95 and 81, whereas the ion at *m/z* 765 gave fragments at *m/z* 537 (M–C_14:0_), 509 (M–C_16:0_), 225, 165, 153, 95 and 81, as indicated in the fragmentation pathway (Figure 4), being consistent with the SQDG structure, esterified by myristic and palmitic acids. Structures of sulfonoglycolipids are frequently studied by tandem-MS, and the fragments at *m/z* 225, 165, 153, 95 and 81can be considered as diagnostics for 6-deoxy-6-sulfono-hexosyl residue [6,28,32,33]. 

The lower abundant ions in the MS^1^ spectrum occurred due to other fatty acids combination, such as that one at *m/z* 793, usually associated with sulfoquinovosyl-dipalmitoylglycerol or myristoyl-steaorylglycerol. Although the structures had been well characterized by MS^2^, the absolute hydroxyl configuration could not be determined by mass spectrometry. Since SQDG (*m/z* 765) was the predominant glycolipid found in Fraction 2, this fraction was used for NMR experiments, such as COSY, TOCSY and HSQC and biological activity as well.

### 2.4. NMR Spectroscopy of Sulfolipids

**Figure 5:**
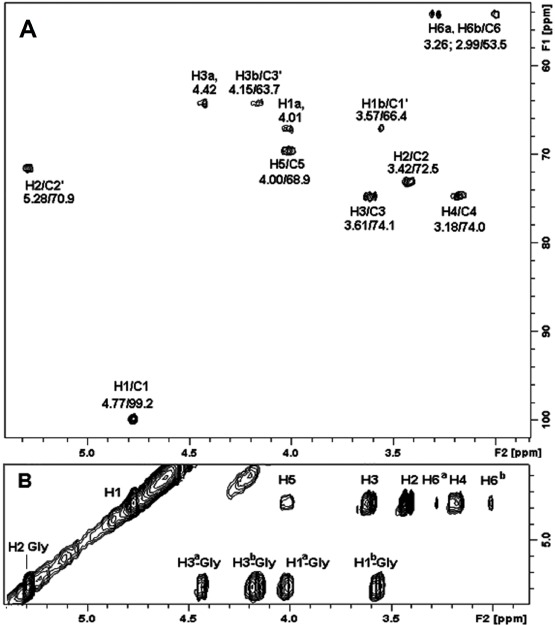
(**A**) Partial 2D ^1^H/^13^C-HSQC fingerprint spectrum of sulfoquinovosyldiacylglycerol in fraction 1, showing the polar region of lipid; (**B**) Partial 2D ^1^H/^1^H-TOCSY spectrum, showing the fingerprint region containing the monosaccharide ring connectivity and glycerol moiety of the sulfonoglycolipid.

The structure of the sulfoglycolipid present in fraction 2, was confirmed by ^1^H and ^13^C NMR analysis, based on HSQC and TOCSY fingerprints [5,6]. The chemical shift map was obtained by HSQC and discussed as follows. The HSQC spectrum of glycolipid contained aliphatic signals from δ 22.8 to 34.6 with predominant CH_2_ signals at δ 29.9 and CH_3_ at δ 14.1. The spectrum suggests the presence of exclusively saturated fatty acids, since no double bond signals were observed at δ 122.0 to 133.0 [6,33] and confirms the previous ESI-MS/MS results. The signals of ^1^H/^13^C-HSQC and ^1^H/^1^H-TOCSY from glycerol were observed at δ 4.009, 3.570/66.4; 5.280/70.9; and 4.417, 4.159/63.5, arising from C-1, C-2, and C-3, respectively (Figure 5A,B).

On the ^1^H/^13^C-HSQC experiment, the anomeric region contained a single signal at δ 4.770/99.2, consistent with α-quinovopyranosyl group. Also, other key ^1^H/^13^C-HSQC signals were found at δ 3.267, 2.990/53.5. The presence of doublets of CH_2_ signals in a high-field region is characteristic of *S*-substituted C-6, typical of 6-sulfo-α-quinovopyranosyl unit [5,6]. In addition, the total hydroxyl connectivity observed in ^1^H/^1^H-TOCSY experiment (Figure 5B) confirmed the hydroxyl configuration of a 6-deoxy-α-glucopyranoyl (α-quinovosyl) unit. These results and those from mass spectrometry were very similar to previous findings for sulfonoglycolipids [4,5,6,7,34,35,36], confirming the lipid identity as 1,2-di-*O*-acyl-3-*O*-(6-deoxy-6-sulfo-α-D-glucopyranosyl)-*sn*-glycerol (SQDG).

### 2.5. Antiviral Activity

In a previous study from our group, the antiviral activity of the crude acetone and methanol fractions from a lipid extract of *O.* *obtusiloba* was evaluated and the results have demonstrated that both fractions had potent inhibitory activity against HSV-1 (herpes simplex virus type 1) and present low toxicity for cell cultures [22]. A better activity was found in the crude methanol fraction. In order to find out which compounds are responsible for this activity, a purified sulfoglycolipid fraction (Fraction 2) was obtained in the present work and the antiviral activity against HSV-1 and HSV-2 (herpes simplex virus type 2) was measured (Table 1).

**Table 1:** Cytotoxicity and antiviral activity of fraction 2 from *Osmundaria obtusiloba* and acyclovir.

			HSV-1			HSV-2		
Compounds	MNTC (µg/mL)	CC50 (µg/mL)	PI	EC50 (µg/mL)	SI	PI	EC50 (µg/mL)	SI
Fraction 2 *O. obtusiloba*	50	72	75	42	1.7	96	12	6.0
Acyclovir	200	>200	99	0.8	>250	99	1.38	>145

HSV-1, herpes simplex virus type 1; HSV-2, herpes simplex virus type 2; MNTC, maximum non-toxic concentration; CC_50_, 50% cytotoxic concentration; PI, percentage of inhibition; EC_50_, effective concentration to reduce virus titers by 50%; SI, selectivity index. Acyclovir, standard compound.

Our results showed a reduction in the antiviral activity of the purified glycolipid (75%) when compared with the crude methanol fraction (99.5%) [22]. The somewhat higher activity of the crude methanol fraction relative to the purified fraction 2 occurred, presumably, as a result of the absence of other polar lipids in this fraction , such as MGDG and DGDG, which can trigger a synergic effect. These results suggest the importance of both glycolipids (glycoglycerolipids and sulfoglycolipids) in the antiviral activity. The anti-HSV activity of glycolipids extracted from seaweed has been demonstrated [19,20,21,22]. El-Baroty and coworkers showed that glycolipid isolated from the brown alga *Dilophys fasciola* was able to block the viral infectivity by interacting with HSV-1 glycoprotein [22]. Studies will be conducted to determine the mechanism of action of sulfoglycolipid from *O. obtusiloba*. DGDG and SQDG from *O. obtusiloba* identified in this present work have been previously detected in other red algae *Hypnea musciformis*, *Porphyra acanthophora*, *Pterocladiella capillacea*, the brown alga *Dictyota menstrualis *and *D. cervicomis *and in the green alga *Caulerpa racemosa* [22], confirming that these glycolipids are conserved molecules in green, red and brown algae isolated from the Southeastern Brazilian coast [22].

## 3. Experimental Section

### 3.1. Biological Material

*Osmundaria obtusiloba* (C. Agardh) R. E. Norris (Rhodophyta-Fam. Rhodomelaceae) was collected at Praia Rasa, (22°44′3.15″S, 41°57′30.15″O), located at the city of Buzios, Rio de Janeiro, Brazil. The seaweed was washed with local sea water and separated from sediments, epiphytes and other associated organisms.

### 3.2. Extraction and Fractionation of Lipids

*Osmundaria obtusiloba* was treated with acetone and kept overnight at −14 °C. The acetone insoluble material was successively extracted at room temperature with chloroform/methanol 2:1 and 1:2 (v/v). Extracts were combined, dried and the crude lipid extract was partitioned according to Folch and coworkers [25]. The lipids recovered from the Folch lower phase were fractionated on a silica gel column eluted with cloroform, acetone and then methanol. The methanol fraction containing the glycolipids was further purified on a silica gel column, which was sequentially eluted with chloroform/methanol with increasing concentrations of methanol (95:5, 90:10, 80:20, 70:30, 50:50, v/v) and finally with 100% methanol. Fractions eluted with chloroform/methanol 70:30 and 50:50 (v/v) yielded partially purified glycolipid fractions F1 to F5. These fractions were analysed by TLC, developed with CHCl_3_:CH_3_OH:2M NH_4_OH (40:10:1 v/v/v) and the spots visualized with iodine and by spraying with orcinol/H_2_SO_4_ [26]. Fraction 2, was further purified by preparative TLC, yielding a purified glycolipid fraction.

### 3.3. Mass Spectrometry

The MS analysis was carried out in an electrospray ionization mass spectrometry (ESI-MS), model Quattro-LC (Waters) with a triple-quadrupole mass analyzer, operating at atmospheric pressure ionization (API), assisted by a syringe pump (KDScientific) for sample infusion. Nitrogen was used as nebulizing and desovation gas and the ionization energies were 50 V on the cone and 2 kV on the capillary, operating in the negative ionization mode or 80 V (cone) and 2.5 kV (capillary) when operating in the positive ionization mode. The second stage tandem-MS was obtained by collision induced dissociation mass spectrometry (CID-MS) using argon as collision gas and collision energies ranging between 35–60 eV. The samples were prepared in MeOH at 1 mg/mL, then diluted to 0.1 mg/mL in MeOH-H_2_O (7:3, v/v) containing 1 mM LiCl, for the positive ion detection, and direct infused into ESI source, at a flow rate of 10 µL/min.

### 3.4. Nuclear Magnetic Resonance

The glycolipid component of F2 was deuterium exchanged by repeated dissolution in MeOD–D_2_O (2:1 v/v) and freeze-drying. The spectra were obtained from solutions of MeOD–CDCl_3_ (1:1) at 30 °C, using TMS (tetramethylsilane) as reference standard (δ = 0). All spectra were obtained with a Bruker 400 MHz AVANCE *III* NMR spectrometer with a 5 mm inverse gradient probe. 2D-NMR experiments were carried out using HSQC, COSY and TOCSY. The experiments were recorded for quadrature detection in the indirect dimension, edited-HSQC spectra were acquired using 128 scans per series of 1 K × 256 W data points with zero filling in F1 (4 K) prior to Fourier transformation [37].

### 3.5. Cells and Viruses

Vero cells (African green monkey kidney) were grown in Eagle’s minimum essential medium (Eagle-MEM) and supplemented with 10% (v/v) fetal bovine serum, glutamine (2 mM), garamycin (50 µg/mL), fungizone (amphotericin B) (2.5 µg/mL), NaHCO_3_ (0.25%) and HEPES (10 mM). HSV-1 was isolated from a typical lip lesion and HSV-2 from a typical genital lesion in the Virology Department of the Federal University of Rio de Janeiro (UFRJ), Brazil. Viruses were typed by polymerase chain reaction (PCR) using specific primers for identification [38].

### 3.6. Cytotoxicity Assay

The cytotoxicity of glycolipids was performed by incubating Vero (African green monkey kidney cell) cell line monolayers cultivated in 96-well microplates with two-fold serial dilutions (200–3.1 µg/mL) of the SQDG (F-2) for 48 h at 37 °C in a 5% CO_2_ atmosphere. Cellular viability was evaluated by the neutral red dye-uptake method [39]. The 50% cytotoxic concentration (CC_50_) was deﬁned as the SQDG concentration which caused a 50% reduction in the number of viable cells.

### 3.7. Antiviral Activity Assay

The antiviral activity of SQDG and acyclovir was evaluated by the titer reduction. The virus titers were calculated using the Reed and Muench statistical method [40] and expressed as 50% tissue culture infective dose (TCID_50_) per mL. Vero cell monolayers were treated with the SQDG and acyclovir at the MNTC and 100 TCID_50_/mL of HSV-1 or HSV-2 suspensions were added to treated and untreated cell cultures and incubated at 37 °C for 48 h in a 5% CO_2_ atmosphere. After incubation, the supernatant was collected and virus titers in treated and untreated cells were determined. The antiviral activity was expressed as percentage of inhibition (PI) [41] using antilogarithmic TCID_50_ values as follows: PI = [1 − (antilogarithmic test value/antilogarithmic control value)] × 100. The dose-response curve was established starting from the MNTC (50–0.78 µg/mL to SQDG and 200–0.2 µg/mL to acyclovir) and the 50% effective concentration (EC_50_) was defined as the concentration required for 50% protection against virus induced cytopathic effects. The selectivity index (SI) was determined as the ratio of CC_50_ to EC_50_.

## 4. Conclusions

SQDG molecules have been isolated for the first time from the red alga *Osmundaria obtusiloba* and their structure elucidated by ESI-MS/MS and ^1^H and ^13^C NMR analysis, based on HSQC and TOCSY fingerprints. MGDG and DGDG have also been identified on the basis of electrospray ionization tandem MS/MS spectrometry. We also demonstrated that SGDG had potent antiviral activity against HSV-1 and HSV-2 and present low toxicity for cell cultures. Although acyclovir has shown better activity, the inhibitory effect in different steps of viral infection is a desirable feature allowing a more efficient action and preventing the emergence of resistance strains. The identification of the chemical structure of algae glycolipids could contribute for a better understanding of the relation between these structures and biological activities.
